# Crucial Role of Increased Arid3a at the Pre-B and Immature B Cell Stages for B1a Cell Generation

**DOI:** 10.3389/fimmu.2019.00457

**Published:** 2019-03-15

**Authors:** Kyoko Hayakawa, Yue-Sheng Li, Susan A. Shinton, Srinivasa R. Bandi, Anthony M. Formica, Joni Brill-Dashoff, Richard R. Hardy

**Affiliations:** Fox Chase Cancer Center, Philadelphia, PA, United States

**Keywords:** B1a, Arid3a, Lin28b, B-1 development, CLL/lymphoma

## Abstract

The Lin28b^+^Let7^−^ axis in fetal/neonatal development plays a role in promoting CD5^+^ B1a cell generation as a B-1 B cell developmental outcome. Here we identify the Let7 target, Arid3a, as a crucial molecular effector of the B-1 cell developmental program. Arid3a expression is increased at pro-B cell stage and markedly increased at pre-B and immature B cell stages in the fetal/neonatal liver B-1 development relative to that in the Lin28b^−^Let7^+^ adult bone marrow (BM) B-2 cell development. Analysis of B-lineage restricted Lin28b transgenic (Tg) mice, Arid3a knockout and Arid3a Tg mice, confirmed that increased Arid3a allows B cell generation without requiring surrogate light chain (SLC) associated pre-BCR stage, and prevents MHC class II cell expression at the pre-B and newly generated immature B cell stages, distinct from pre-BCR dependent B development with MHC class II in adult BM. Moreover, Arid3a plays a crucial role in supporting B1a cell generation. The increased Arid3a leads higher Myc and Bhlhe41, and lower Siglec-G and CD72 at the pre-B and immature B cell stages than normal adult BM, to allow BCR signaling induced B1a cell generation. Arid3a-deficiency selectively blocks the development of B1a cells, while having no detectable effect on CD5^−^ B1b, MZ B, and FO B cell generation resembling B-2 development outcome. Conversely, enforced expression of Arid3a by transgene is sufficient to promote the development of B1a cells from adult BM. Under the environment change between birth to adult, altered BCR repertoire in increased B1a cells occurred generated from adult BM. However, crossed with B1a-restricted V_H_/D/J IgH knock-in mice allowed to confirm that SLC-unassociated B1a cell increase and CLL/lymphoma generation can occur in aged from Arid3a increased adult BM. These results confirmed that in fetal/neonatal normal mice, increased Arid3a at the pre-B cell and immature B cell stages is crucial for generating B1a cells together with the environment for self-ligand reactive BCR selection, B1a cell maintenance, and potential for development of CLL/Lymphoma in aged mice.

## Introduction

The RNA-binding protein Lin28 is highly expressed during embryogenesis and disappears by adulthood in human and mouse (1). Lin28b expressing fetal hematopoietic stem cells (HSC) are capable of supporting the B-1 B cell development in mice, but this capacity is lost in adult bone marrow (BM) HSC. Specifically, Lin28b^+^Let7^−^ cells progressed to the B cell progenitor stage (pro-B) in fetal/neonatal liver, in contrast to Lin28b^−^Let7^+^ pro-B cell in adult bone marrow (BM) (2, 3). In mice, this early Lin28b^+^Let7^−^ fetal/neonatal B cell development, as B-1, allows increased generation of CD5^+^ B cells, termed B1a, with autoreactive/polyreactive BCRs, in contrast to adult Lin28^−^Let7^+^ B-2 development (3, 4). A portion of fetal/neonatal generated mouse B1a cells self-renew throughout life and play predominantly positive roles in innate immunity (5, 6). However, in aged mice, B1a cells with restricted BCRs can spontaneously become chronic lymphocytic leukemia (CLL)/lymphoma cells or accelerate CLL/lymphoma generation by expressing the T-cell leukemia 1 (TLC1) oncogene (7, 8). While Lin28b plays a critical role in supporting B-1 development through its repressive effects on Let7 family micro-RNAs (mir), the downstream targets through the Lin28b^+^Let7^−^ axis remain to be elucidated. We strongly considered the possibility that the DNA-binding protein Arid3a might play a role (3). Arid3a mRNA bears consensus Let7 and mir-125b binding sites and both of these miRNAs are absent in Lin28b^+^ fetal/neonatal liver progenitors. Thus, Arid3a expression is induced in fetal pro-B cells relative to that in adult pro-B cells. Transduction of Arid3a shRNA in fetal pro-B cells resulted in loss of B1a cell generation. In contrast, transduction of adult BM pro-B cells with Arid3a-expressing retrovirus generated B1a cells (3). Thus, Arid3a may be both necessary and sufficient for B1a cell development.

Arid3a, also known as Bright in mouse and Dril1 in human, is a member of the AT-rich interaction domain superfamily of DNA binding protein (9, 10). Arid3a is the prototypical Arid3 member and is involved in large chromatin modulating complexes. Arid3 members play roles in embryogenesis and Arid3a is ubiquitously expressed by various tissues in the embryo (11, 12). In embryonic stem cells (ES), Arid3a plays an important role in regulating whether the ES cell will renew or differentiate. Arid3a expression is low in self-renewing ES cells, and then is dramatically up-regulated upon ES cell differentiation, where it serves as a key regulator of cell-fate decisions by suppressing ES lineage plasticity and maintaining the differentiated state (11, 13–15). After birth, Arid3a expression becomes restricted to B cells and myeloid cells, including erythrocytes (10, 16). In the B-lineage in fetal/neonatal liver, Arid3a is expressed at the pro-B cell stage (3), and as shown here, further strongly increased at the pre-B and immature B cell stages, compared to in adult BM, then decreased in mature B cells.

Arid3a was originally named B cell regulator of immunoglobulin heavy chain transcription, Bright (9). Arid3a/Bright binds to nuclear matrix association regions in certain IgH V_H_ promoter proximal sites, together with Bruton's tyrosine kinase Btk and Btk-mediated phosphorylated TFII-1, promotes immunoglobulin transcription, and increases B lymphocytes (17). Specifically, Bright was originally found to promote the IgH V_H_S107, which encodes the T15 anti-phosphorylcholine (aPC) BCR and the use of this aPC T15 BCR is restricted to B1a cells (18). Arid3a's ability to promote the generation of particular IgHs requires its interaction with Btk, and mutated Btk with a loss of Btk role in Xid mice abrogates the generation of B1a cells (19). Notably, in addition to IgH-increasing role for Arid3a with Btk and TFII-1 in the nucleus, Arid3a shuttles between the nucleus and cytoplasm, where Arid3a localizes with the BCR in lipid rafts within the plasma membrane, where it interacts with Btk in the BCR signaling complex (20, 21). Importantly, the presence of high levels of Arid3a in lipid rafts raises the threshold of Btk-dependent BCR signaling, by decreasing Btk activation (21). Since Arid3a plays role in both increasing autoreactive BCRs such as T15 B1a cells and regulating their signaling capacity, we considered that the appropriately increased Arid3a expression observed in Lin28b expressing fetal/neonatal precursors is required for the initial B-1 development with B1a cell generation in normal mice.

Whether Arid3a is the key gene in B-1 development with B1a cell generation was unclear. The elimination of several genes involved in BCR signaling has resulted in the loss of B1a cell generation (22). It was also found that loss of I-type Siglec-G lectin (sialic acid-binding immunoglobulin-like lectin G) or C-type CD72 lectin, both negative regulators of BCR signaling, resulted in increased B1a cell generation (23, 24). In contrast, loss of the HLH (helix-loop-helix) transcription factors Myc (cMyc) or Bhlhe41 (Dec2/Sharp1) downregulated B1a cell generation (25, 26). Furthermore, MHC class II expression is different between B-1 and B-2 development. MHC class II is negative at the pre-B and immature B cell stages on d0-d5 after birth in liver, but positive in adult BM (27, 28). Arid3a is the highest at the pre-B and immature B cell stages in neonatal mice. Based on these observations, it is important to know that differences in Arid3a levels at the pre-B and immature B cell stages between B-1 and B-2 development contribute to alterations in expression levels of these genes, resulting in distinct B cell development outcomes. Further, another difference between B-1 and B-2 development is at the pre-BCR stage, since expression of IgH without surrogate light chain (SLC) association can generate B1a cells from B-1 development, but not from B-2 development (29). Thus, a critical role for Arid3a in the generation of BCR without SLC has also to be confirmed. Here, to explore this pathway, we analyzed B-lineage restricted Lin28b Tg mice (Rag2-Lin28b-λ5LCR Tg), Arid3a knockout mice (Arid3a KO-CD2Cre), and Arid3a Tg mice (Rag2-Arid3a-PolyA Tg). To assess the susceptibility of these mice to CLL/lymphoma generation upon aging, these mouse lines were also crossed with Eμ-hTCL1 Tg mice. Since Lin28b Tg mice appropriately increased Arid3a in adult BM, we further crossed Lin28b Tg mice with B1a-restricted V_H_/D/J IgH knock-in mice V_H_11t (V_H_11KI) and ON25 (V_H_Q52KI) (8, 30), since these V_H_11 (V11-2)/D/J_H_1 and V_H_Q52 (V2-9)/D/J_H_4 IgHs do not associate with SLC (8, 31). Our data confirmed that increased Arid3a at the pre-B and immature B cell stages is key for B1a cell generation in B-1 development, including without the requirement of SLC.

## Results

### Increased B1a Cells in Lin28b Transgenic Mice

**Figure 1:**
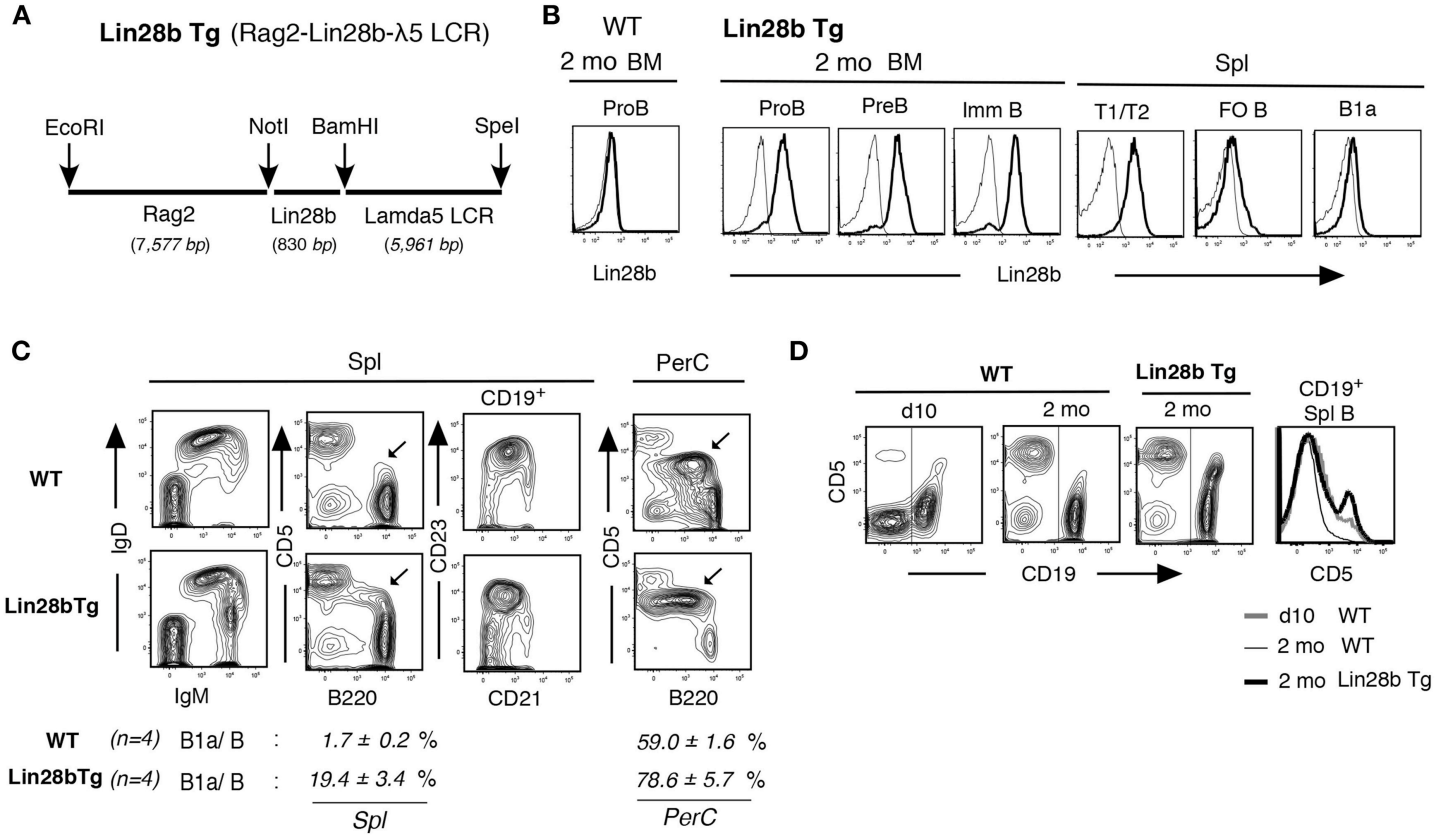
Increased B1a cells in Lin28b Tg mice. **(A)** Rag2-Lin28b-λ5 LCR construct for B lymphoid restricted Lin28b Tg expression. **(B)** Cytoplasmic staining of 2 mo Lin28b Tg (and WT) BM B-lineage and spleen transitional T1/T2, FO B, and B1a cells. Rabbit anti-Lin28b + anti-rabbit Ab staining (high black), vs. anti-rabbit alone (–) as a control. WT mouse B-lineage and B cells were all Lin28b negative. **(C)** 2 mo adult Lin28b Tg and WT mouse spleen and PerC. B220^lo^CD5^+^ B1a cells are marked. B1a/B; percentage of B1a cells in total B cells. B1a cell increase was also observed in Lin28b Tg.B6 (C57BL/6) mice. **(D)** CD5 level comparison in spleen B cells (CD19^+^, marked) between WT (d10 and 2 mo) and Lin28b Tg (2 mo) mice.

To generate a tractable experimental system in which to assess the molecular basis by which the Lin28b^+^Let7^−^ axis regulates B-1 development, we engineered transgenic (Tg) mice expressing Lin28b in B lymphoid progenitors under the control of the Rag2 promoter and the λ5 surrogate light chain locus control region (LCR) (Figure 1A). Introduction of this Tg enforced Lin28b protein expression in adult mice from B cell-committed CD19^+^ Pro-B through the immature B cell stage in the BM and in transitional (T1/T2) B cells in the spleen, as confirmed by cytoplasmic staining (Figure 1B). In adult Lin28b Tg mice, B220^lo^CD5^+^ B1a cells (with IgM^hi^IgD^med/lo^) were increased in spleen, and dominant in peritoneal cavity (PerC) (Figure 1C). In normal wild type (WT) mice, CD5 expression in newly generated B1a cells in spleen of neonatal mice was higher than in B1a cells in adult mice. CD5 expression was also higher in adult Lin28b Tg mice, similar to the neonatal B1a cells (Figure 1D).

### Lin28b Tg^+^ Pre-B and Immature B Cells in Adult BM Resemble Normal Mouse Neonatal Liver

**Figure 2:**
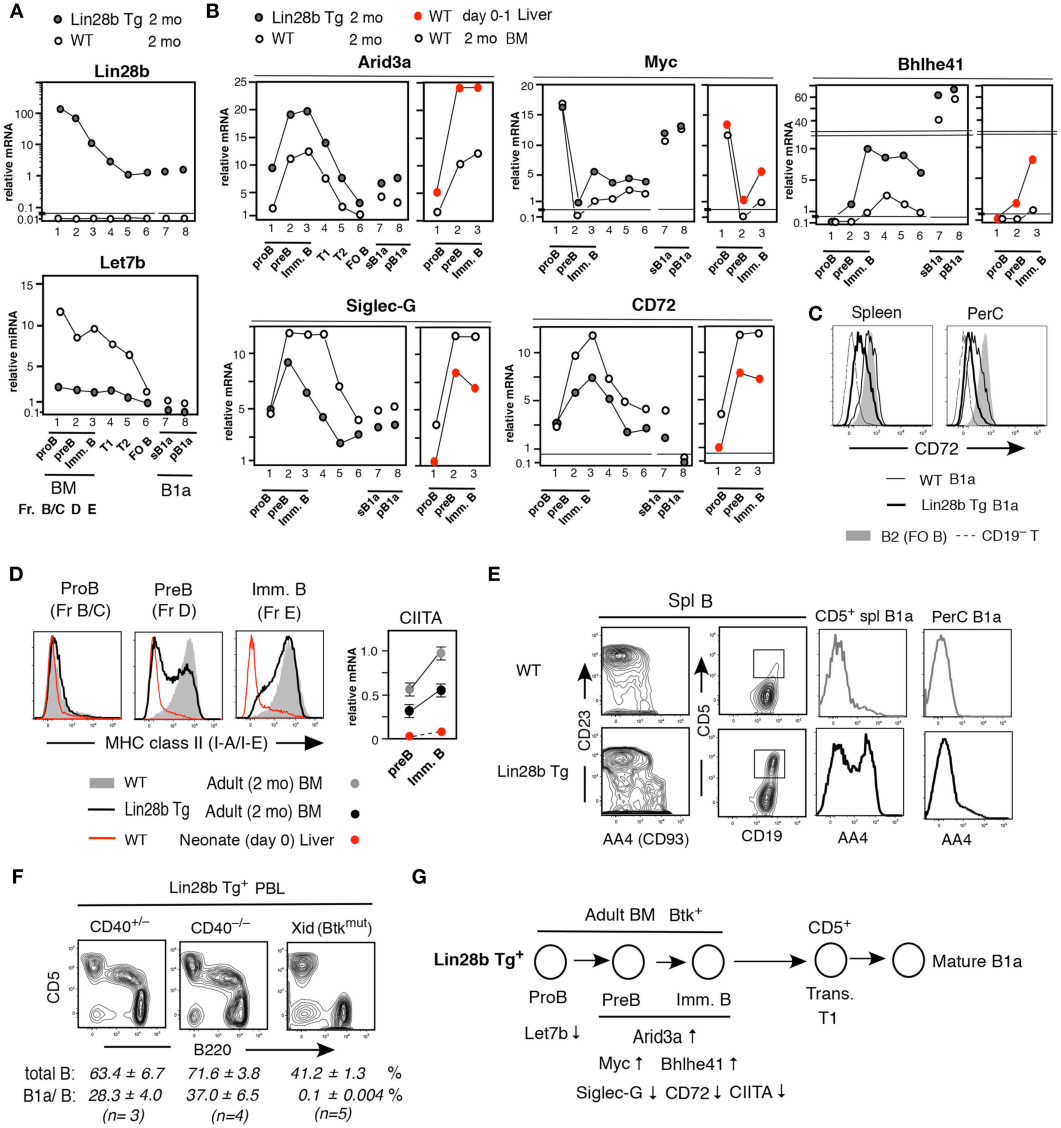
Pre-B and immature B cells in adult Lin28b Tg BM resembles WT fetal/neonatal liver and generate B1a cells. **(A,B)** qRT-PCR. Adult (2 mo) Lin28b Tg mouse B lineage in BM, B cells in spleen, and B1a cells in spleen (sB1a) and in PerC (pB1a), in comparison to WT mice. **(B)** Addition of qRT-PCR of WT mouse neonatal (d0-1) liver vs. adult BM B-lineage. The results shown represent the mean of three independent determinations. Samples under the same mouse background showed similar mRNA levels (and mirRNAs). **(C)** CD72 staining of WT and Lin28b Tg mouse B1a cells in spleen and PerC, and WT B2 cells (FO B cells in spleen). **(D)** Left; MHC class II staining of pro-B, pre-B, and immature B cells in 2 mo WT and Lin28b Tg BM, and WT d0 liver. Right; CIITA qRT-PCR. *n* = 3 each; mean ± s.e. **(E)** Comparison of AA4^+^ transitional stage in spleen B cells. AA4 level in CD19^+^CD5^+^ B cells in spleen (square region) and PerC, in Lin28b Tg and WT mice. **(F)** PBL analysis of 2 mo Lin28b Tg mice crossed with CD40 KO mice, and with Xid mice. Total B; CD19^+^, B1a/B; B220^lo^CD5^+^B in total B. **(G)** Forced expression of Lin 28b Tg in adult BM led to the indicated gene expression changes in pre-B and immature B cells, resembling that of fetal/neonate mice, and increasing the ability to generate B1a cells.

Lin28b represses the processing of let-7 microRNAs (1). In Lin28b Tg mice, adult B-lineage cells showed elevated levels of Lin28b mRNA and reduced Let7b miRNA, as expected (Figure 2A), together with increased Arid3a mRNA (Figure 2B). In WT mice, Arid3a expression is elevated in neonatal liver B-lineage cells relative to that in adult BM, with expression increasing during differentiation from pro-B cell stage through the pre-B and immature B cell stages (Figure 2B, right side in red). Likewise, the expression of Myc and Bhlhe41 mRNAs is also higher in neonatal pre-B and immature B cells in WT mice, whereas expression of Siglec-G and CD72 mRNAs is lower than adult BM (Figure 2B, right side in red). In mature B1a cells present in WT mice, Myc and Bhlhe41 mRNAs are further increased, and Siglec-G and CD72 mRNAs are further decreased than in pre-B and immature B cell stages (Figure 2B, left side). These B-1 development associated changes in gene expression in WT mice appear to be regulated by Lin28b, as they were replicated in adult BM and B cell outcome from Lin28b Tg mice (Figure 2B, left side). For CD72, the Lin28b Tg actually further reduced protein expression even than observed in normal B1a cells in the PerC (Figure 2C). MHC-class II (I-A/I-E) expression also differs between B-1 and B-2 B-linage development. In WT mice, on day0 (to day5) after birth, surface MHC class II protein is negative in early B-lineage, including at the IgM^+^ immature B cell stage in liver, and MHC class II transactivator CIITA (32) is low (Figure 2D, red). In contrast, in adult BM, MHC class II expression starts to occur at pre-B cell stage together with increased CIITA (Figure 2D). This neonatal pattern for MHC-class II in WT mice was mimicked in adult Lin28b Tg mouse BM pre-B and immature B cells associated with reduced expression of the MHC class II and CIITA (Figure 2D, black). Consistent with the Lin28b Tg orchestrating changes in gene expression linked to the B-1 development program, the Lin28b Tg also increased the generation B1a cells in adult mice, as evidenced by the greater proportion of AA4/CD93^+^ immature B1a cells present in spleen in adult Lin28b Tg mice, while most B1a cells in the PerC were mature AA4^−^ B cells (Figure 2E), as observed for normal B1a cells found in d0-10 neonatal WT mice (33). Importantly, the increase in CD5^+^ B1a cells observed in spleen, including in peripheral blood (PBL), from adult Lin28b Tg mice was independent of CD40 signaling, but was dependent on Btk (Figure 2F), as expected for normal B-1 derived B1a cell generation (7, 19). Thus, ectopic expression of Lin28b in adult B-lineage precursor cells remodeled the gene expression patterns in pre-B and newly generated immature B cells to resemble that of normal B-1 development in neonatal mice with the high capacity to generate B1a cells (summarized in Figure 2G).

### Arid3a Deficiency Attenuates B1a Cell Generation and Leads to Adult-Type B Cell Development

**Figure 3:**
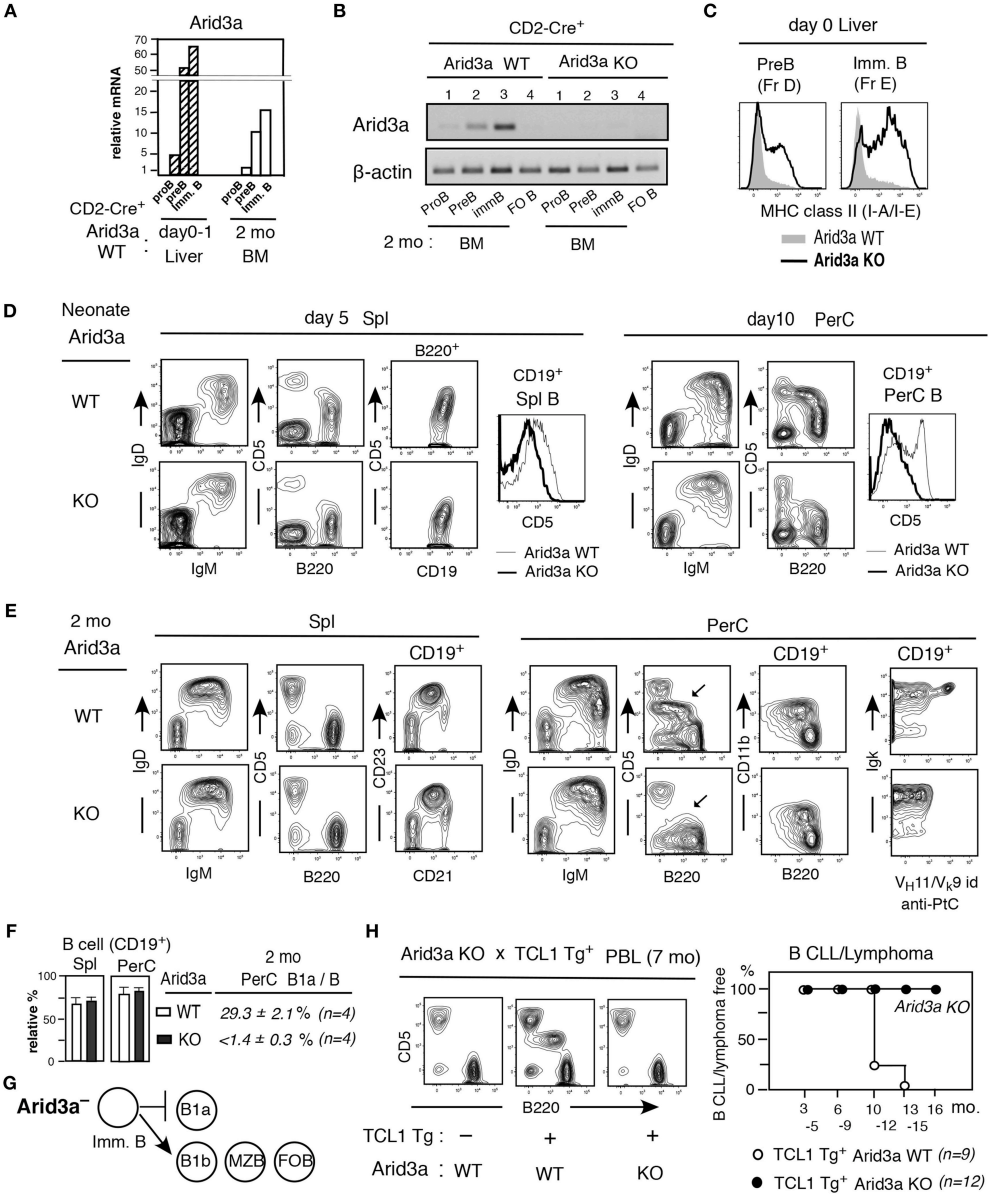
Arid3a KO mice exhibit adult-type B cell development without B1a cell generation. **(A)** Arid3a qRT-PCR of B-lineage in CD2-Cre^+^ Arid3a WT mice in d0-1 liver and 2 mo BM. *n* = 2 each; mean. **(B)** Arid3a PCR of 2 mo BM B-lineage and spleen FO B cells in CD2-Cre^+^ Arid3a WT and KO littermates. **(C)** MHC class II staining of pre-B and immature B cells in d0 Arid3a KO and WT littermates. Represent of 3 mice each. All showed similar increases in Arid3a KO compared to WT. **(D)** Neonatal spleen (day 5) and PerC (day 10) analysis of Arid3a KO and WT littermates, and CD5 level comparison in B cells (CD19^+^). Three mice each showed similar results. **(E,F)** Adult (2 mo) Arid3a KO and WT spleen and PerC analysis. B1a cells in PerC are marked. Percentage of total B cells in spl and PerC in lymphoid area (*n* = 4 each; mean ± s.e.) and B1a cells in total B cells in PerC. **(G)** Arid3a deficient mouse generates B1b, MZ B, and FO B cells, and loss of B1a cell generation. **(H)** PBL crossed with TCL1 Tg-CD2-Cre^+^ mice. CLL/lymphoma incidence difference in TCL1 Tg expressing Arid3a KO vs WT littermates.

We speculated that increased Arid3a in Lin28b Tg^+^ mice plays a key role in affecting expression of genes required for B1a cell generation. To assess this, we next analyzed Arid3a knockout mice (Arid3a KO). Arid3a KO mice were crossed with CD2-Cre mice, both in the C57BL/6 background (Figure S1). In CD2-Cre^+^Arid3a WT mice, Arid3a mRNA was elevated in neonatal Pre-B and immature B cells than in the same stages from adult BM as in normal C.B17 mice (Figure 3A). In contrast, in CD2-Cre^+^Arid3a KO mice, RT-PCR analysis revealed that Arid3a KO effectively eliminated Arid3a expression from adult BM B-lineage (Figure 3B). Arid3a expression is low in splenic FO B cells in WT, as reported previously (16) (Figure 3B). Arid3a-deficiency caused a marked increase in MHC class II protein expression in neonatal pre-B and immature B cells, as is observed in adult B-2 BM, suggesting that Arid3a loss was perturbing the neonatal gene expression pattern (Figure 3C). On neonatal day5, splenic B cells in Arid3a KO mice were predominantly IgM^+^IgD^hi^, including a more prominent IgM^lo^IgD^hi^ population likely to become FO B cells (Figure 3D, left). Moreover, Arid3a-deficiency also prevented the upregulation of CD5 on splenic B cells (Figure 3D, left). These effects were more pronounced in the PerC on neonatal day10 (Figure 3D, right). In adult mice, the absolute number of B cells in spleen and PerC was unchanged by Arid3a-deficiency (Figures 3E,F), and there was no change in the representation of the FO B and MZ B cell populations in the spleen (Figure 3E). In contrast, CD5^+^ B1a cells were completely absent from the PerC of adult Arid3a KO mice (Figures 3E,F) as previously found (34), including those expressing the B1a restricted V_H_11^+^ anti-PtC (phosphatidylcholine) BCR normally found in WT mice (Figure 3E, right). Distinct from CD5^+^CD11b^+^ B1a cell loss, CD5^−^ CD11b^+^ B1b cells were present in the PerC by Arid3a-deficiency (Figure 3E), consistent with the Btk-independence of B1b cell development (35). Thus, Arid3a-deficiency selectively abrogated the B1a potential of fetal/neonatal B cell progenitors, while fully preserving their potential to support B-2 B cell development, indicating that Arid3a-deficiency switched the B-1 developmental potential of fetal/neonatal liver to that resembling adult B-2 BM development (summarized in Figure 3G). When crossed with TCL1 Tg mice, Arid3a KO mice still showed no increased B1a cells in PBL during aging, and B CLL/lymphoma incidence did not occur, in contrast to Arid3a WT littermates (Figure 3H). Thus, the attenuation of B1a development by Arid3a-deficiency blocked the ability to promote development of B CLL/lymphoma.

### Enforced Expression of Arid3a Promotes the Development of MHC Class II Negative Pre-B and Immature B Cells in Adult BM and Continued B1a Cell Presence

**Figure 4:**
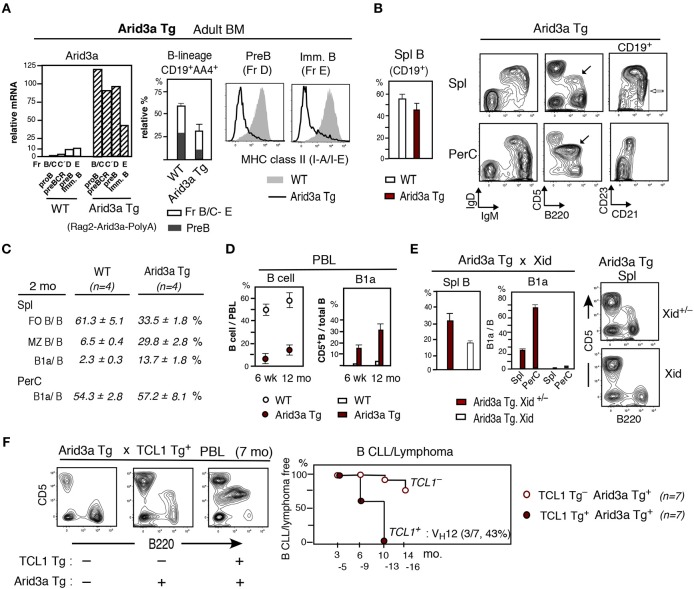
Arid3a Tg mice show reduction of adult BM B cell generation and B1a cell CLL/lymphoma generation in aged mice. **(A)** Adult Arid3a Tg (Rag2-Arid3a-PolyA)^+^ and Arid3a Tg^−^ (WT) mouse BM. Left: qRT-PCR of 2 mo adult B-lineage (including pre-BCR, Fr.C'). *n* = 2 each; mean. Middle: relative total CD19^+^AA4^+^ B-lineage (without large myeloid cells) and pre-B cell percentages in 2–4 mo BM. *n* = 4 each; mean ± s.e. Right: MHC class II staining of pre-B and immature B cells. Representative of 2–4 mo 4 mice each (Arid3a Tg mice were all negative). 2 mo Arid3a Tg.B6 mice also showed decreased total B-lineage and MHC class II in BM. **(B)** Left: Spleen B cell (CD19^+^) percentage in adult (2–4 mo) Arid3a Tg and WT mice. *n* = 4 each; mean ± s.e. Right: 2 mo Arid3a Tg mouse spleen and PerC. B1a cells are marked (black) and MZ B cells are marked (white). **(C)** Percentage of FO B, MZ B and B1a cells in total B cells. **(D)** PBL B cell percentage, and B1a cells in total PBL B cells of Arid3a Tg^+^ and Arid3a Tg^−^ (WT) mice at 6 wk and 12 mo. *n* = 6 each; mean ± s.e. **(E)** Arid3a Tg mice crossed with Xid mice. 2 mo Arid3a Tg^+^ littermates. *n* = 3 each; mean ± s.e. Xid = homozygous Btk^Xid^ **(F)** Arid3a Tg mice crossed with TCL1 Tg mice, and PBL analysis. High CLL/lymphoma incidence in TCL1 Tg^**+**^ (including aPtC V_H_12^+^ BCR lymphoma) vs. TCL1 Tg^−^ Arid3a Tg mice.

To determine if enforced expression of Arid3a could confer B1a cell potential on adult BM, we generated Arid3a Tg mice in which an Arid3a was expressed under the controls of the Rag2 promoter and SV40 PolyA site (Rag2-Arid3a-PolyA). In adult BM, the Arid3a Tg markedly increased the expression of Arid3a, with the highest level occurring in the CD19^+^ pro-B cell stage, also high at pre-BCR stage, and Arid3a remained to be higher at the pre-B and immature B cell stages as compared to WT adult BM (Figure 4A). Strongly elevated Arid3a expression altered the B cell developmental potential in adult BM in that total B-lineage cells (CD19^+^AA4^+^) were decreased, including reductions in generation of pre-B (Figure 4A, middle) and also immature B cells. Distinct from Arid3a deficient mice, the pre-B and immature B cells present in this Arid3a Tg BM exhibited strongly reduced MHC class II expression (Figure 4A, right), similar to what is observed in early neonatal liver in WT mice. As expected from the reductions of B-lineage in the BM of Arid3a Tg mice, B cells in the spleen were also reduced (Figure 4B, left), including decreased FO B cells (CD21^med^CD23^+^AA4^−^) (Figures 4B,C). Notably, both MZ B cells (CD21^hi^CD23^lo/−^AA4^−^) and B1a cells were increased within reduced B cells in spleen relative to non-Arid3a Tg (WT) control mice. B1a cells in PerC of Arid3a Tg was dominant, being comparable to that in WT mice (Figures 4B,C). The B cell content of peripheral blood (PBL) was also reduced in Arid3a Tg mice. However, circulating B1a cells were increased with age (Figure 4D). The capacity of ectopically expressed Arid3a Tg in B1a cells by adult BM was dependent upon Btk, as their generation was abrogated upon crossing the Arid3a Tg mice to Xid mice, in which Btk is inactivated, including in spleen (Figure 4E). Enforced expression of Arid3a also augmented the capacity of the TCL1 Tg to promote B CLL/lymphoma, including those expressing the V_H_12^+^ aPtC BCR as previously found among the B1a cell-derived B CLL/lymphoma in TCL1 Tg^+^ mice (7) (Figure 4F). Thus, strongly enforced expression of Arid3a at the pro-B and pre-BCR stages in adult BM caused a general reduction in B cell development. However, it confirmed that overexpression of Arid3a in adult BM directed MHC class II negative pre-B and immature cell generation similar to early neonatal liver, and Btk dependent B1a cells were continuously present, increased in aged, and capable of becoming CLL/lymphoma.

### Differences in Arid3a Levels Lead to Distinct Pre-B and Immature B Cell Outcomes

**Figure 5:**
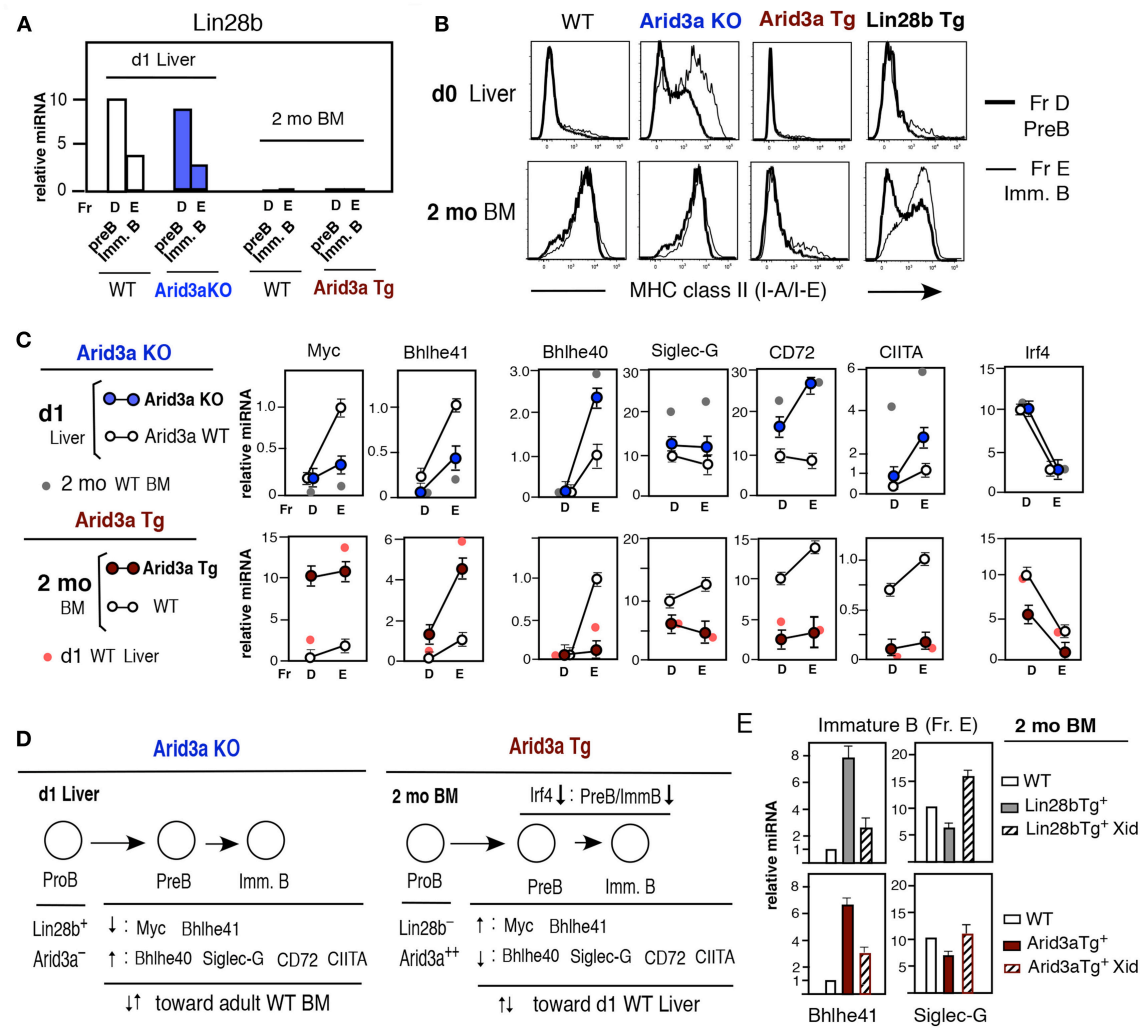
Altered Arid3a level impacts levels of various mRNAs at the pre-B and immature B cell stages in neonatal liver and adult BM. **(A)** Lin28b qRT-PCR of pre-B and immature B cells in d1 liver Arid3a KO and Arid3a WT mice, and 2 mo BM Arid3a Tg^+^ and Tg^−^ WT mice. Two to three mice each. Lin28b expression in d1 liver and lack in adult BM were all similar. **(B)** MHC class II staining of d0 liver and 2 mo BM pre-B and immature B cells in WT, Arid3a KO, Arid3a Tg, and Lin28b Tg mice. **(C)** qRT-PCR of pre-B and immature B cells. d1 liver; Arid3a KO mice (*n* = 3, mean ± s.e.) and Arid3a WT mice (*n* = 2, mean ± s.e.) and addition of 2 mo normal mouse (WT C57BL/6) BM (*n* = 2, mean) for comparison. 2 mo BM; Arid3a Tg (*n* = 4, mean ± s.e.) and Arid3a Tg^−^ (WT) (*n* = 3, mean ± s.e.), and addition of d1 normal mouse liver (*n* = 2, mean). **(D)** Summary of alteration of Arid3a KO d1 liver mRNAs and Arid3a Tg 2 mo BM mRNAs. **(E)** Lin28b Tg and Arid3a Tg mice were crossed with Xid mice. Bhlhe41 and Siglec-G mRNA comparison in immature B cells (Fr. E) with or without Xid, and normal WT immature B cells as a control, in 2 mo BM mice. *n* = 3 each; mean ± s.e.

The capacity of Arid3a loss or overexpression to modulate B1a cell development was not caused by altered expression of Lin28b, since Lin28b mRNA levels in Arid3a-deficient and Tg mice were similar to WT mice, including at the pre-B and immature B cell stages (Figure 5A). To assess the extent to which Arid3a-deficiency or overexpression directly influenced the gene expression program in fetal/neonatal B-1 and adult B-2 B cell development, we examined effects on the expression of genes differentially expressed in neonatal vs. adult pre-B and immature B cells. Arid3a-deficiency resulted in premature upregulation of MHC-II in neonatal liver, as is typically observed in adult BM, and its ectopic expression by Arid3a Tg in adult BM repressed MHC- class II, replicating a fetal/neonatal expression pattern, more than Lin28b Tg mice (Figure 5B). Arid3a loss and overexpression similarly altered the expression patterns of other genes comprising a signature typifying B1a development. These include Myc, Bhlhe41, Bhlhe40, Siglec-G, CD72, and CIITA (Figure 5C), which function in modulating B1a cell development. Arid3a KO mice in neonatal d1 liver showed decreased Myc and Bhlhe41 mRNAs, while Bhlhe40, Siglec-G, CD72, and CIITA mRNAs were increased (Figure 5C). In contrast, Arid3a Tg mice in adult BM showed increased Myc and Bhlhe41 mRNAs, and decreased Bhlhe40, Siglec-G, CD72, and CIITA mRNAs (Figure 5C). Myc and Bhlhe41 deficiency was known to decrease B1a development, Siglec-G and CD72 deficiency increases B1a development, and CIITA regulates the expression of MHC class II (23, 24, 32). Bhlhe41 negatively regulates Bhlhe40 expression, providing an explanation for their reciprocal expression pattern (36). The modulation of these genes during neonatal and adult development and their regulation by Arid3a is summarized in Figure 5D. In normal mouse adult BM, interferon regulator factor Irf4 plays a major role in generating pre-B cells from the pre-BCR stage (37, 38), but is dispensable for fetal/neonatal B cell development (39). The repression of Irf4 by enforced expression of Arid3a in adult BM (Figure 5C, bottom right) may be responsible for the reduction in adult B-2 cell development distinct from WT BM. The changes in gene expression induced by enforced expression of Arid3a in adult BM partly involved Btk dependence, since the increase in Bhlhe41 and the decrease in Siglec-G mRNA levels caused by Arid3a overexpression were reversed by the loss of Btk function in Xid mice, as also found for Lin28b induced changes in gene expression (Figure 5E). Similar effects were observed on Myc expression (Figure S2). However, this was not the case for CD72 (Figure S2), suggesting that its regulation is Btk independent. Thus, the alterations in expression of most Arid3a target genes were dependent upon Arid3a–Btk association and also independent on Btk at the pre-B and immature B cell stages before Btk dependent ligand-induced BCR signaling activation occurred to become CD5^+^ B1a cells. Arid3a level plays a crucial role in distinct fetal/neonatal B-1 and adult B-2 B cell developmental outcomes.

### Generation of B1a Cells With SLC-unassociated BCR and B1a Cell Tumor Development From Adult BM in Lin28b Tg Mice

**Figure 6:**
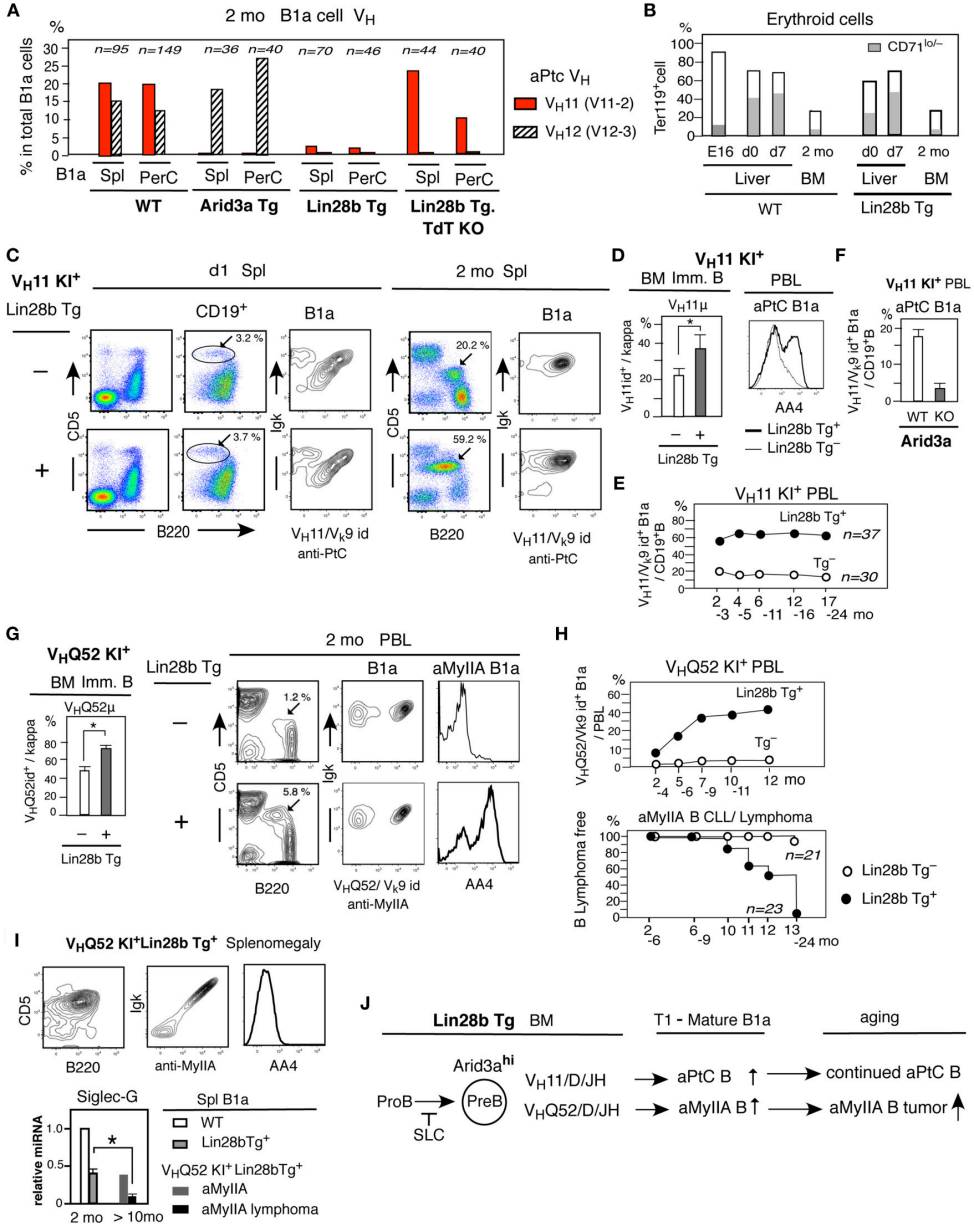
SLC unassociated BCR B1a cell generation from adult Lin28b Tg BM and B1a lymphoma generation in aged mice. **(A)** Single cell sequence data of B1a cells in spleen and PerC in 2 mo WT (C.B17), Arid3a Tg, Lin28b Tg, and Lin28b Tg with TdT KO mice, and percentage of V_H_11^+^ and V_H_12^+^ B cells. **(B)** Percentage of erythroid cell (Ter119^+^) together with matured erythrocytes (Ter119^+^CD71^lo/−^) in WT and Lin28b Tg^+^ mice. WT mice; liver *n* = 2 each, 2 mo BM *n* = 5, mean. Lin28b Tg^+^ mice; liver and 2 mo BM *n* = 3 each, mean. **(C)** Lin28b Tg mice crossed with V_H_11 KI mice. B1a cell frequency in d1 and 2 mo spleen (marked), and V_H_11^+^aPtC cell dominance in B1a cells. **(D)** Left; 2 mo BM V_H_11μ^+^ cell percentage in immature B cells in V_H_11 KI mice with or without Lin28b Tg. *n* = 3 each; mean ± s.e. ^*^P < 0.05. Right; AA4 staining of aPtC B1a cells in PBL (2 mo). **(E)** V_H_11^+^ aPtC B1a cell percentage in PBL B cells with or without Lin28b Tg. Mean of Lin28b Tg^+^ mice (*n* = 37) and Lin28b Tg^−^ mice (*n* = 30) origin. **(F)** V_H_11 KI.B6 mice crossed with mice to generate Arid3a KO and WT. V_H_11^+^ aPtC B1a cells in PBL B cells in 2 mo Arid3a KO (*n* = 4) and WT (*n* = 5) littermates; mean ± s.e. **(G)** Lin28b Tg mice crossed with V_H_Q52 KI mice. Left; 2 mo BM V_H_Q52μ^+^ cell percentage in immature B cells in V_H_Q52 KI mice with or without Lin28b Tg. *n* = 3 each; mean ± s.e. ^*^P < 0.006. Right; 2 mo PBL. B1a cell percentage (marked) with aMyIIA and AA4 staining. **(H)** Up; aMyIIA B1a cell frequency in PBL in V_H_Q52 KI mice with Lin28b Tg^+^(*n* = 23) and Tg^−^ mice (*n* = 21) origin. Bottom; aMyIIA B1a cell CLL/lymphoma incidence. **(I)** Spleen in 14 mo V_H_Q52 KI^+^Lin28b Tg^+^ mouse with splenomegaly. Bottom; Siglec-G qRT-PCR of spleen B1a cells. 2 mo WT and Lin28b Tg. (*n* = 3 each) and >10 mo non-lymphoma (*n* = 1), and lymphoma (*n* = 3) aMyIIA B1a. mean ± s.e. ^*^P < 0.016. **(J)** Arid3a involvement in adult Lin28b Tg BM to increase SLC unassociated aPtC and aMyIIA autoreactive B1a cell generation.

The B-2 development in adult BM requires pre-BCR stage associated with surrogate light chain (SLC) to generate the pre-B cell stage (37, 40). In contrast, fetal/neonatal B-1 development includes B cell generation without the SLC-associated pre-BCR stage (29, 31). We wished to determine whether an increased Arid3a in adult BM allows generation of B1a cells with SLC-unassociated BCR. Anti-PtC V_H_11/V_k_9-128(br9) and V_H_12/V_k_4-91(kf4) BCRs are commonly found among B1a cells in WT adult mice (Figure 6A) originally generated from B-1 development. Notably, while V_H_12 IgH does associate with SLC, V_H_11 IgH fails to do so (29, 31) (Figure S3), thus, V_H_11^+^ B1a cells are the SLC-unassociated outcome as previously known (31). In contrast to this V_H_11- and V_H_12- increased B1a cells in WT mice, the V_H_12 BCR was highly represented but the V_H_11 BCR was depleted in Arid3a Tg B1a cells, and both V_H_11 and V_H_12 BCRs were not well represented among B1a B cells from Lin28b Tg mice (Figure 6A). Thus, BCR repertoire alteration in B1a cells occurred in Arid 3a increased adult mice, with SLC- unassociated V_H_11^+^ BCR decrease. BCR repertoire alteration in B1a cells including with negative V_H_11 was also found in increased B1a cells in Siglec-G deficient mice (41).

However, when Lin28b Tg mice were crossed to mice deficient for TdT (terminal deoxynucleotidyl transferase), the incidence of V_H_11 IgH was increased under TdT^−^ (Figure 6A). TdT is an enzyme that adds non-templated nucleotides to recombination junctions, generating N-addition at V-D and/or D-C, allowing increased various BCR repertoire. In normal mice, TdT is negative/low in fetal/neonate liver and high in adult BM (42, 43). As previously found in Lin28b-GFP^+^ mouse BM (3), Lin28b Tg mice down-regulated TdT (*Dntt*) in adult BM B-lineage than adult WT mice, but still expressed TdT, being higher TdT than fetal/neonatal B-lineage. SLC-unassociated V_H_11 IgH in B1a cells is without N-addition (44), being TdT-independent, and V_H_11^+^ BCR increased in TdT deficient Lin28bTg^+^ mice. Thus, we considered that it is possible that increased BCR repertoire occurred from adult Arid3a-increased BM due to TdT^+^, together with differences in environmental ligand availability to in the fetal/neonatal liver vs. the adult BM. V_H_11^+^ and V_H_12^+^ aPtC BCRs are both reactive with erythrocytes with digested membrane phosphatidylcholine (44, 45). Ter119^+^ erythroid cells including mature CD71^lo/−^ erythrocytes are less prevalent in adult BM than in fetal/neonatal liver in both WT and B-lineage restricted Lin28b Tg mice (Figure 6B), also in Rag2-dependent Arid3a Tg mice. Thus, the altered BCR repertoire present in the adult Arid3a Tg and Lin28b Tg B1a cells may be resulted from differences in the environment (e.g., ligand availability) between birth to adult, with increased competitive BCRs from TdT^+^ adult BM.

Thus, to determine whether SLC-unassociated BCR generation can occur from Arid3a increased adult BM, Lin28b Tg mice were crossed with V_H_11/D/J knock-in mice V_H_11t (V_H_11 KI) in the context of reduced competitive BCR generation. On day 1 after birth, although a variety of different BCRs were generated in Lin28b Tg V_H_11 KI mice, the early developmental stages of B1a cell maturation in the spleen were dominated by usage of V_H_11^+^ aPtC BCRs, as previously reported for non-Tg mice (33) (Figure 6C). In adult Lin28b Tg mice, further increased V_H_11^+^ aPtC BCR B1a cells were observed than in Lin28b Tg^−^ mice (Figure 6C). Initially generated immature B cells in BM showed increased V_H_11μ^+^ expression in Lin28b Tg^+^ mice, and circulating immature and mature V_H_11^+^ aPtC B1a cells in PBL were consistently higher as the outcome of continuous generation from BM, including in aged mice (Figures 6D,E). V_H_11 KI mice crossed with Arid3a KO mice showed strongly decreased V_H_11 aPtC BCR B1a cell generation (Figure 6F). Thus, Arid3a is involved in increasing the prevalence of this SLC^−^V_H_11^+^ BCR in B1a cells in the context of Lin28b Tg mice.

To further assess whether the increase in B1a cells caused by ectopic activation of the Lin28b/Arid3a in adult BM can develop spontaneous tumors with SLC^−^ IgH BCRs, we next crossed V_H_Q52/D/J knock-in mice ON25 (V_H_Q52 KI) with Lin28b Tg mice. The V_H_Q52 (V2-9) IgH does not associate with SLC, lacks N-additions at the V-D-J junctions, and generates B1a cells expressing the V_H_Q52/V_k_9-96(ce9) BCR. This BCR is reactive with non-muscle myosin IIA (aMyIIA) and exhibits strong reactivity to MyIIA^+^ platelets (8). The Lin28b Tg resulted in an increase in V_H_Q52μ^+^ immature B cells in adult BM and in circulating immature aMyIIA B1a cells (Figure 6G). The prevalence of these cells increased further in aged mice, resulting in splenomegaly in the majority of aging Lin28bTg^+^ V_H_Q52KI mice as compared to Lin28b Tg^−^ (Figure 6H). These aMyIIA B1a cells ultimately transformed into malignant cells with the characteristic of mature cells, with decreased Siglec-G expression (Figure 6I). The reduced Siglec-G expression may contribute to pathogenesis, since Siglec-G deficiency increases B-cell lymphoproliferative disorders (46). Thus, the increased Arid3a in Lin28b^+^Let7^−^ outcome at the pre-B cell stage allowed the generation of SLC-unassociated autoreactive BCR B1a cells from adult BM, and ultimately support the ability to develop B1a cell tumors (summarized in Figure 6J).

## Discussion

Here we report that Arid3a serves as a critical downstream target of Lin28b^+^Let7^−^ signaling, which is both necessary and sufficient to support the B1a cell generation. Arid3a is highly expressed in pro-B cells and further increasing at the pre-B and immature B cell stages in fetal/neonatal liver as a B-1 development process to generate B1a cells. SLC-dependence difference is the early B-1 vs. B-2 development distinction before B1a cell generation. While adult Lin28b^−^Let7^+^ B-2 development requires pre-BCR signaling with SLC association, fetal/neonatal Lin28b^+^Let7^−^ induced B-1 development can occur in the absence of pre-BCR signaling (29). Autoreactive BCR IgHs, including V_H_11 and V_H_Q52 (V2-9), fail to associate with SLC, yet support B-1 development with B1a cell generation. V_H_S107 (V7-1) IgH, originally found as an Arid3a/Bright target with B1a cell generation, exhibits low binding to SLC (Figure S3). Several CLL/lymphoma BCRs found in mice were also SLC-unassociated IgHs (7, 8). These IgHs are restricted to B-1 derived B cells. The SLC independence of BCRs raises the question of how the BCR signals that promote development are generated. Pax5 is a critical transcription factor that suppresses the T cell fate by repressing Notch1 transcription, and activates expression of CD19 and SLP-65 to initiate a B cell gene expression program. Pax5 allows V_H_-DJ_H_ recombination and binds to the VpreB and λ5 promoters to generate surrogate light chain (SLC) (47, 48), leading to expression of the SLC in developing B-1 cells. However, since Arid3a can promote the transcription of certain IgHs (not all IgHs) and Pax5 promotes Jκ transcription (48), both IgH and Igκ will be generated, resulting in early expression of BCRs that likely substitute for the pre-BCR and directly promote pre-B to immature B cell development in fetal/neonate mice. HLH transcription factor E2A is known to be critically involved in the initial D_H_-J_H_ rearrangement before B-lineage generation, and E2A also rearranges V_k_-J_k_ to generate Igκ (49). We found that the increased Arid3a expression induced by our Lin28b Tg promoted the development of SLC-independent V_H_11 or V_H_Q52 KI progenitors into B1a B cells in adult BM. Conversely, Arid3a deficiency blocked the development of V_H_11^+^ aPtC B1a cells. Thus, in normal mice, increased Arid3a by Lin28b^+^Let7^−^ plays a major role in allowing Pre-BCR- independent B cell development.

After the initial generation of immature B cells from pre-B cells, the development of CD5 expressing B1a cells results from activation of BCR signaling through recognition of the self-ligands in the environment. This is facilitated by the reduced expression of Siglec-G and CD72 by neonatal B-1 development. Upon BCR interaction with ligand, the Src family tyrosine kinase Lyn is rapidly phosphorylated and activates BCR signaling. However, Lyn activation is dampened by the actions of Siglec-G/CD72–SHP-1 (24, 50, 51). In this pathway, activated Lyn phosphorylates the immunoreceptor tyrosine-based inhibitory motifs (ITIM) in Siglec-G and CD72, which recruit and activate SHP-1 phosphatase, leading to inhibition of B cell activation. As originally discovered, SHP-1 deficient motheaten mice exhibited a marked increase in B1a cell development (52), and both Siglec-G and CD72 deficiency lead to increased Ca^2+^ signaling and NFATc1 activity, and more CD5^+^ B1a cells in the spleen and PerC (23, 24). Here, we find that Arid3a reduces Siglec-G and CD72 expression, which is likely one way that it promotes autoreactive B1a cell development. Moreover, Arid3a shuttles between the nucleus and cytoplasm, and can interact with the BCR complex in lipid rafts, suggesting that it may also play a more proximal role in regulating BCR signaling (21). One key signaling molecule of the BCR complex is Btk, which binds both Arid3a and Lyn kinase (53). The capacity of Arid3a to repress Siglec-G is impaired by Btk inactivation. Thus, increased Arid3a is likely to facilitate B1a B cell generation by relieving the inhibition of BCR signaling caused by negative regulators like Siglec-G and CD72.

Arid3a also controls the expression of DNA-binding proteins, like the HLH transcription factors Myc and Bhlhe41. After increased at the immature B cell stage, Myc remains elevated in mature B1a cells (7), and Bhlhe41 becomes the highest in B1a cells but not in FO B and MZ B cells (26). The increased expression of Myc and Bhlhe41 is important for maintaining autoreactive B1a cells. Like Arid3a, Bhlhe41 is diversely expressed through embryonic development. Bhlhe41(Sharp1/Dec2) regulates the differentiation of several cell types, and represses transcriptional activation, and promotes cell survival, but not proliferation (36, 54, 55). In B cells, Bhlhe41 also represses genes encoding cell-cycle machinery, thereby controlling B cell proliferation (26). Bhlhe41 interacts with Bhlhe40, a transcriptional activator, and can impair Bhlhe40 promoter induction, thus negatively regulating Bhlhe40 expression (36). Since Bhlhe40 strongly represses cell proliferation, leading to growth arrest, and terminal differentiation (56), negative regulation of Bhlhe40 by Bhlhe41 is likely to further contribute to B1a cell persistence. Since Bhlhe41 can directly repress CD72 (26), Bhlhe41 induction by Arid3a may help to repress CD72 and promote B1a BCR signaling and continue to maintain Bhlhe41^hi^CD72^low^ B1a cells present in PerC (Figures 2B,C). In Bhlhe41^lo^ B-2 development, Bhlhe41 is further downregulated from the transitional stage in spleen to generate Bhlhe41^−^ mature B cells, but after activation, plasma cells express Arid3a and Bhlhe41 (57), possibly to control these differentiated B cells. In T cells, Bhlhe41 is mostly not expressed, however, GATA3 induces Bhlhe41/Dec2 expression and provides an important role in generating CD4^+^ T_H_2 cells (2). Thus, both Arid3a and Bhlhe41 are continuously playing roles throughout life. In neonatal stage, Arid3a plays important roles in B cell development. Increased Arid3a in pre-B and immature B cells decreases the negative regulators of BCR signaling, Siglec-G and CD72, and increases Myc and Bhlhe41, thereby allowing self-ligand reactive BCR signal induced B1a cell generation and maintenance with self-renewal throughout life.

Lin28 disappears in adult. As this outcome, Lin28^−^Let7^+^ Arid3a^lo^ B-2 development critically involves the pre-BCR with SLC to pre-B cell transition stage. Pax5 is continuously important to promote adult B cell development as in the fetal/neonatal stage, which correlates with expression of SLP-65. After the SLC combined pre-BCR is generated, pre-BCR-induced proliferation occurs through phosphoinositide-3 kinase (PI3K) signaling. FoxO1 and Irf4 directly bind to Pax5 which induces SLP-65, and SLP-65 down-regulates the initial pre-BCR PI3K activity to block proliferation to allow progression to the pre-B cell stage (37). Irf4 synergizes with the Ets family transcription factor PU.1, as Irf4-PU.1 complex, which enhances Igκ light chain rearrangement, and also activates CIITA, leading to MHC class II expression (58, 59). Thus, the presence of Irf4 and PU.1 is an important transitional switch from the pre-BCR proliferation stage to generate a pre-B cell with Igκ rearrangement, different from fetal/neonatal B-1 development not requiring Irf4 and PU.1 (39, 60). In the absence of Arid3a, the generation of MHC class II^+^ pre-B and immature B cells occurs in fetal/neonates, and adult B-2 development is normal. In converse, increased Arid3a in adult BM of Arid3a Tg mice decreased Irf4 expression and resulted in lower B-lineage generation with MHC class II negative pre-B and immature B cells. Thus, Arid3a^lo^ in WT and Arid3a^−^ in Arid3a KO leads to the pre-BCR to pre-B-dependent process, distinct from Arid3a^hi^.

Increased Arid3a in adult by transgene allowed B1a cell generation. However, BCR repertoire in B1a cells was distinct from WT due to environment difference between neonate and adult, and increased BCR repertoire ability by TdT in adult to generate ligand-induced B1a cells. As found by crossed with V_H_11and V_H_Q52 knock-in mice, normal mouse B1a cell BCR increase in adult Arid3a^hi^ B1a cells can occur under the low increase of competitive B1a cell repertoire. In normal mice, B1a cell generation is decreased in adult by Arid3a^lo^ outcome. Thus, continuous self-renewal and maintenance can occur by early generated B1a cells from B-1 development. In conclusion, Arid3a plays a key role in distinguishing between fetal/neonatal B-1 and adult B-2 development. In fetal/neonatal stages, increased Arid3a at the pre-B cell and immature B cell stages as a Lin28^+^Let7^−^ outcome is crucial for generating B1a cells together with the environment for initial self-ligand reactive BCR selection, B1a cell maintenance, self-renewal throughout life, and the potential for development of CLL/lymphoma in aged mice.

## Materials and Methods

### Generation of Lin28b and Arid3a Transgenic Mice

In order to express Lin28b gene in B lymphoid progenitors, we used a mouse recombination activating gene 2 (Rag2) promoter element consisting of about 7.5 kb fragment upstream of the Rag2 coding region, and a surrogate light chain locus control region (61), to generate a Rag2-Lin28b-λ5LCR construct. The 7.5 kb Rag2 regulatory element was amplified from BAC clone, RP23-178k1, using the following primer pair and cloned into the TA vector. Rag2-EcoRI-forward: ACGTGAATCCTCCTCCCTTCCTCCGACTAT. Rag2-NotI-reverse:ACGTGCGGCCGCTTT TGATTGTGAATAGGTCTTTTATC. Then the Rag2 fragment was released from TA using EcoRI and NotI. The mouse Lin28b full cDNA sequence (830 bp) was digested from Lin28b in pQCXIP vector using NotI and BamHI. The mouse lambda 5 LCR fragment (5.9 kb) was amplified from the BAC clone using primers flanked by BamHI and SpeI-KpnI, cloned into TA vector and then released from TA with BamHI and Spel. Then Rag2, Lin28b, and λ5 LCR were ligated into pBluscript II vector at EcoRI and SpeI sites. The full Rag2-Lin28b-LCR construct was released for transgene injection using KpnI. All construct elements were verified by sequencing. For Rag2-Arid3a-PolyA generation, 7.5 kb Rag2 regulatory element was amplified as described above. The mouse Arid3a full cDNA sequence (1,806 bp) was amplified from mouse cDNA using PCR with NotI and SpeI restriction sites attached at each end. Then Rag2 and Arid3a were ligated into pBlusecript II vector at EcoRI and SpeI sites. SV40 polyA signal sequence was inserted into the 3' end of Arid3a cDNA at SpeI and SacII sites.

### Arid3a Knockout Mice With CD2-Cre

Arid3a knockout mice B6N(Cg)-Arid3a^tm1b(KOMP)Wtsi^, conditional Arid3a allele mice Arid3a^tm1c(KOMP)Wtsi^, and CD2-Cre transgenic mice B6.Cg-Tg(CD2-cre), were purchased from the Jackson Laboratory. These mice were all homozygous and C57BL/6 (B6) background. First, we initiated continuous breeding of Arid3a tm1b mice with CD2-Cre^+^mice, and Arid3a tm1c mice with CD2-Cre^+^ mice. To obtain Arid3a KO mice, Arid3a tm1b^+^Cre^+^ mice were crossed with Arid3a tm1c^+^Cre^+^ mice. Within littermates from crossing, PCR screening was used to identify tm1b^+^tm1c^+^CD2Cre^+^ mice lacking the wild type Arid3a primer site as the Arid3a KO-CD2Cre^+^ mouse, and the tm1b^−^tm1c^+^CD2Cre^+^ mice with wild type Arid3a primer site as Arid3a WT CD2-Cre^+^ mice, as a control (Figure S1). To generate TCL1 Tg^+^ Arid3a KO and V_H_11KI^+^ Arid3a KO mice, Tg (or KI) mice under the C57BL/6 background were first crossed with the CD2-Cre mice, then Tg^+^ (or KI^+^) with Cre^+^ mice were crossed with tm1b^+^Cre^+^ mice to obtain Tg^+^(or KI^+^) tm1b^+^Cre^+^mice, followed by crossing with tm1c^+^Cre^+^ mice, to obtain Tg^+^(or KI^+^) Cre^+^ Arid3a KO mice.

### Mice

Lin28b Tg (Rag2-Lin28b- λ5 LCR Tg) and Arid3a Tg (Rag-2-Arid3a-PolyA Tg) mouse lines were crossed with C.B17 mice, and also crossed with C57BL/6J mice. V_H_/D/J_H_ knock-in mouse lines, V_H_11t (V_H_11 KI) and ON25 (V_H_Q52 KI), were originally C.B17 mice background as previously described, and also backcrossed with C57BL/6 mice. CD40 KO mice were C.B17 background and Btk mutant Xid mice and TdT KO mice were BALB/c background. Eμ-hTCL1 Tg mouse line, TCL1 Tg, was originally under C57BL/6 background, and also backcrossed on the C.B17 mice as previously described (7). The majority of data with Lin28b Tg and Arid 3a Tg experiments were from mice on the C.B17 background, with confirmation by analysis of mice under the C57BL/6 background as described in Figure legend. Arid3a KO vs WT mice were on the C57BL/6 background, and crossed with TCL1 Tg C57BL/6 mice, and also with V_H_11 KI C57BL/6 mice.

### Flow Cytometry Analysis, Sorting, and Reagents

Multicolor flow cytometry analysis, sorting, and monoclonal antibody reagents, including anti-idiotype (id) antibodies for anti-V_H_11id (3H7), anti-PtC V_H_11/V_k_9id (7H11), anti-V_H_Q52id (13F11), and anti-MyIIA V_H_Q52/V_k_9id (24E1) have been described previously (8, 33). FL-anti-MHC class II (I-A/I-E), PE-anti-CD71, and FL-anti-CD72. Cytoplasmic Lin28b protein level comparison between adult Lin28b Tg vs. WT mice were done by rabbit anti-mouse Lin28b together with Alexa647 goat anti-rabbit.

### B-lineage Sorting From Liver and BM and MHC Class II Analysis

For the sorting of B-lineage from fetal/neonatal liver and adult BM for qRT-PCR analysis, selection markers of early B-lineage cells (CD19^+^B220^+^AA4^+^, CD11b/Mac1^−^Gr1^−^, LybC^−^Ter119^−^CD3^−^) together with CD43^+^IgM^−^CD24^med^ for pro-B (Fr. B/C), CD43^+^IgM^−^CD24^hi^ Pre-BCR (Fr. C'), CD43^−^IgM^−^CD24^hi^ pre-B (Fr. D), and IgM^+^IgD^−^ immature B (Fr. E) were used. For sorting of B-lineage from neonatal liver, neonatal d0 livers were used for pro-B, and d0-1 neonatal livers for pre-B and immature B sorting. For MHC class II expression analysis, I-A/I-E was used together with CD19, AA4, CD43, IgM, and IgD staining.

### Quantitative RT-PCR Assay

Gene expression was quantitated by real-time PCR, using TaqMan assays from Applied Biosystems, an ABI 7,500 real-time thermal cycler, and ABI software (Life Technologies). Relative gene expression levels were normalized using β-actin values for mRNA and sno202 for miRNA as a standard.

### Single Cell B1a Cell V_H_/D/J_H_ Sequence Analysis

The procedure for single cell sequencing was previously described (3, 7). V_H_ and V_k_ gene nomenclature list in the C.B17 mouse was described previously (7), with addition of IMGT (www.imgt.org) nomenclature.

### B Cell Leukemia/Lymphoma Diagnosis

PBL analysis was performed every 2-3 months for each mouse. The mice showing a predominance of B1a (CD19^+^B220^lo^CD5^+^) cells in PBL were followed by tissue analysis. B cell neoplasia mostly associated with splenomegaly with B1a cell dominance was defined as lymphoma, with or without significant increase of total PBL cell number, as CLL/lymphoma.

### Erythroid Cell Analysis

For analysis of erythoid cell percentage in embryonic/neonatal liver adult BM, pre-treatment with ammonium chloride was not involved. Cells were stained with Ter119 and CD71 antibodies together with CD19, AA4, CD5 antibodies. In CD19^−^AA4^−^CD5^−^ cells, Ter119^+^ cells as total erythroid cells and Ter119^+^ CD71^lo/−^ cells as maturing erythrocytes.

## Data Availability

The datasets generated for this study can be found in Arid3a, 7811.

## Ethics Statement

Experiments using animals were conduced under approved by the FCCC Institutional Animal Care and Use Committee (IACUC).

## Author Contributions

RH designed constructs to generate Lin28 Tg and Arid3a Tg mice. Y-SL made these Tg mouse lines. SS analyzed originally generated lines. SB did Lin28b cytoplasmic staining. After these original analyses, together with Lin28b and Arid3a mRNA analysis, KH decided to select each Lin28 Tg and Arid3a Tg mouse line for further analysis and wrote this manuscript. SS continued to maintain these Tg mouse lines, selected Arid3a KO and WT mice, and crossed with various mouse lines. SS also performed B-lineage sorting, qRT-PCR, PCR, and immunoglobulin sequence. AF performed IgH-SLC analysis. JB-D contributed to maintain V_H_11KI and V_H_Q52 KI mice and PBL analysis.

### Conflict of Interest Statement

The authors declare that the research was conducted in the absence of any commercial or financial relationships that could be construed as a potential conflict of interest.
