# Supplementary material for: Crucial Role of Increased Arid3a at the Pre-B and Immature B Cell Stages for B1a Cell Generation
Source: Front Immunol. 2019 Mar 15;10:457. doi: 10.3389/fimmu.2019.00457 (PMC6428705; doi:10.3389/fimmu.2019.00457)
Supplement: Supplementary file 1 [file Data_Sheet_1.PDF]

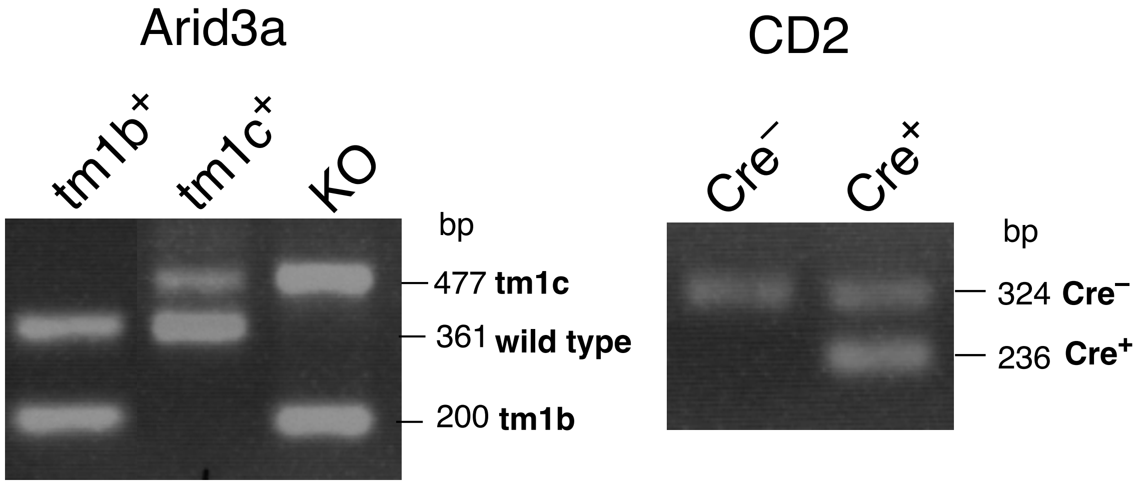

Figure S1. Arid3a KO screening PCR. Arid3a tm1b$^+$Cre$^+$ mouse crossed with Arid3a tm1c$^+$Cre$^+$ mouse. Tim1b$^+$tm1c$^+$Cre$^+$ with lack of wild type Arid3a is the Arid3a KO mouse.

Arid3a
Mutant primers
Forward: ATCCGGGGGTACCGCGTCGAG
Reverse: ACCCCTTCTTCTTTGCCTTC

Wild type primers
Forward: TGTACGTTCATGGTGCCTGT
Reverse: CCTCTTGTCTCCTGTGTGGAG

CD2
CD2-Cre
Forward: AGATGCCAGGACATCAGGAACCTG
Reverse: ATCAGCCACACCAGACACAGAGCAC

CD2-Cre internal positive control
Forward: CTAGGCCACAGAATTGAAAGATCT
Reverse: GTAGGTGGAAATTCTAGCATCATCC
