# Supplementary material for: Crucial Role of Increased Arid3a at the Pre-B and Immature B Cell Stages for B1a Cell Generation
Source: Front Immunol. 2019 Mar 15;10:457. doi: 10.3389/fimmu.2019.00457 (PMC6428705; doi:10.3389/fimmu.2019.00457)
Supplement: Supplementary file 2 [file Data_Sheet_2.PDF]

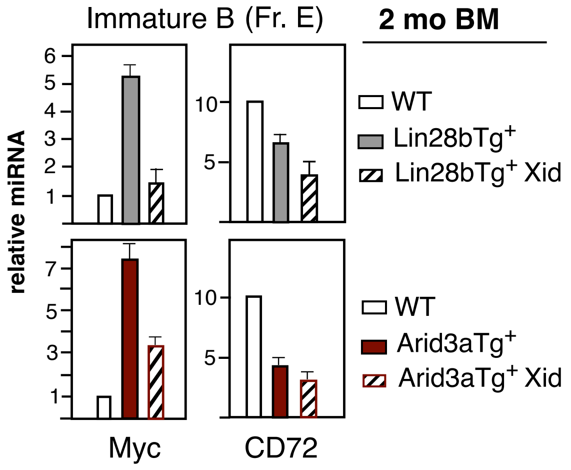

Figure S2. Alteration of increased Myc and reduced CD72 at immature B cell stage in Lin28b Tg and Arid3a Tg mice under the Xid. qRT-PCR. At adult BM immature B cell stage, both Lin28b Tg and Arid3a Tg mice showed increased Myc and decreased CD72 than normal mice (WT). When these Tg were crossed with Btk mutant Xid mice, Myc showed decrease toward WT mice levels. In contrast, CD72 showed further decrease. n=3 each; mean ± s.e.
