# Supplementary material for: Crucial Role of Increased Arid3a at the Pre-B and Immature B Cell Stages for B1a Cell Generation
Source: Front Immunol. 2019 Mar 15;10:457. doi: 10.3389/fimmu.2019.00457 (PMC6428705; doi:10.3389/fimmu.2019.00457)
Supplement: Supplementary file 3 [file Data_Sheet_3.PDF]

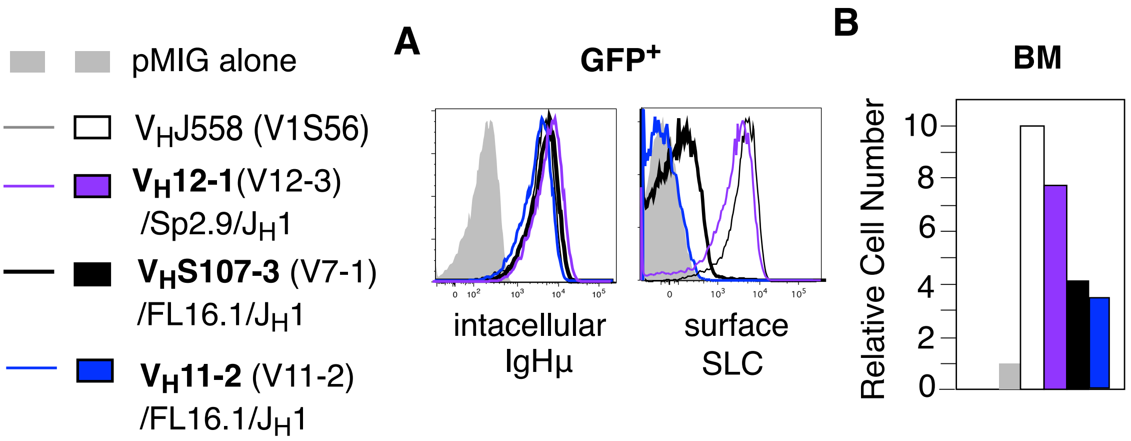

Figure S3. Ability of surrogate light chain (SLC) association by V_H12, V_H11, V_HS107-3 μ in B1a cell. (A) Intracellular IgM level (left) and surface SLC level (right) of GFP^+ cells. A pro-B cell line was transduced with IgH-pMIG retroviral supernatants (GFP^+). pMIG alone was the control (gray), and V_HJ558 (V1S56, SP6) IgH was a positive control. SLC association was $V_H12\text{-}1^+$ (positive), $V_HS107\text{-}3^{lo}$ (low), and $V_H11\text{-}1^-$ (negative). (**B**) BM preBCR-mediated proliferation. B cell numbers from pMIG alone (-) set to 1, and V_HJ558-μ to 10. $n = 3$.

Materials & Methods.

IgH with SLC association analysis. Previously described (*Leukemia* 39:1510, 2016).

(1) *IgH retrovirus production.* Selected IgH VDJ segments were amplified by PCR, cloned into an IgH-μ construct, and inserted into the pMIG retroviral vector (MSCV-IRES-GFP). Each IgH-pMIG plasmid and the pCL Eco retroviral packaging vector were co-transfected into the Phoenix packing line and 24 hour supernatant was collected, filtered, and stored at -80°C until use.

(2) *SLC association analysis*. To analyze Ig heavy chain association with SLC, The N38 Abelson line generated from C.B17.scid was transduced with each IgH-pMIG construct. 2-3 hours post-transduction, supernatant was replaced with complete RPMI-1640. 24 hours after transduction, cells were collected and tested by flow cytometry for IgH-μ expression and pre-BCR assembly, using the conformation-dependent anti-SLC monoclonal antibody SL156 (Yoshikawa et al, *Int. Immunol.* 21:43, 2008) SLC association was calculated as the ratio of surface SL156 level to GFP^+ IgH-μ mean fluorescence intensity. Control heavy chain (SP6-Cμ) was set to 1.0 and used as a positive control.

(3) *Proliferation assay*. Pro-B cells from $Rag^{-/-}$ BALB/c bone marrow were sorted onto pre-established OP-9 stromal cell layers in wells of a 24-well plate and cultured in medium containing recombinant mouse IL-7. The next day, IgH-pMIG retroviral supernatant was added to each well and the plate “spinfected” for an hour. After three hours, supernatant was replaced by fresh complete medium containing IL-7. Three days later, B lineage cells were enumerated by flow cytometry, staining for CD19 and gating $CD19^+$ cells for GFP (IgH-μ) expressio
